# Development and testing of the KERNset: an instrument to assess the quality of telephone triage in out-of-hours primary care services

**DOI:** 10.1186/s12913-017-2686-1

**Published:** 2017-12-02

**Authors:** Marleen Smits, Ellen Keizer, Paul Ram, Paul Giesen

**Affiliations:** 1 Radboud University Medical Center, Radboud Institute for Health Sciences, Scientific Center for Quality of Healthcare (IQ healthcare), P.O Box 9101, 114 IQ healthcare, 6500 HB Nijmegen, The Netherlands; 2 Maastricht University, Faculty of Health Medicine and Life Sciences, Maastricht, The Netherlands

**Keywords:** Quality, Triage, Instrument, Primary care, After-hours

## Abstract

**Background:**

Telephone triage is a core but vulnerable part of the care process at out-of-hours general practitioner (GP) cooperatives. In the Netherlands, different instruments have been used for assessing the quality of telephone triage. These instruments focussed mainly on communicational aspects, and less on the medical quality of triage decisions. Our aim was to develop and test a minimum set of items to assess the quality of telephone triage.

**Methods:**

A national survey among all GP cooperatives in the Netherlands was performed to examine the most important aspects of telephone triage. Next, corresponding items from existing instruments were searched on these topics. Subsequently, an expert panel judged these items on importance, completeness and formulation. The concept KERNset consisted of 24 items about the telephone conversation: 13 medical, ten communicational and one regarding both types. It was pilot tested on measurement characteristics, reliability, validity and variation between triagists. In this pilot study, 114 anonymous calls from four GP cooperatives spread across the Netherlands were judged by three out of eight raters, both internal and external raters.

**Results:**

Cronbach’s alpha was .94 for the medical items and .75 for the communicational items. Inter-rater reliability: complete agreement between the external raters was 45% and reasonable agreement 73% (difference of maximally one point on the five-point scale). Intra-rater reliability: complete agreement within raters was 55% and reasonable agreement 84%. There were hardly any differences between internal and external raters, but there were differences in strictness between individual raters. The construct validity was confirmed by the high correlation between the general impression of the call and the items of the KERNset. Of the differences within items 19% could be explained by differences between triage nurses, which means the KERNset is able to demonstrate differences between triage nurses.

**Conclusions:**

The KERNset can be used to assess the quality of telephone triage. The validity is good and differences between calls and between triage nurses can be measured. A more intensive training for raters could improve the reliability.

## Background

Telephone triage of help requests is often used to manage the workflow in healthcare settings, such as out-of-hours general practitioner (GP) cooperatives [1]. During telephone triage in Dutch GP cooperatives, the level of urgency and required type of healthcare are determined: telephone advice, consultation or home visit with a GP, or referral to the emergency department or ambulance care. In the Netherlands, telephone triage is performed by triage nurses. They use a triage tool, the Netherlands Triage Standard (NTS) [2, 3], and are supervised by GPs.

Telephone triage is a core but vulnerable part of the care process: the assessment is made without visual input and a balance has to be found between efficiency (giving patients the lowest effective level of care) and safety (identifying patients in need of immediate care) [4, 5]. Previous research on the adequacy of telephone triage has shown that on average 10% of all telephone triage contacts are potentially unsafe because of an underestimation of the level of urgency or required type of care [6–9]. In studies using high-risk simulated patients this was even higher: 50%. [5, 6, 10] About half of the patients receiving telephone advice by a triagist eventually have a follow-up contact (47%); the probability of a follow-up contact is lower for patients with more positive experiences with the triage nurse [11]. Of all patient safety incidents in Dutch GP cooperatives, one third are related to telephone triage [12].

Medical knowledge and communication skills of triage professionals are essential to make adequate triage decisions and patient management. The quality of the communication has been found to be positively associated with the appropriateness of the assessment of urgency and required care and thus with safety of triage [4].

In the Netherlands, different instruments have been used to assess the quality of telephone triage at GP cooperatives. The instruments focus mainly on communicational aspects and less on the medical quality of the triage decisions. Moreover, GP cooperatives use different procedures for sampling and rating. The quality of the triage conversations, expressed as the percentage of the maximum score, shows large variation in the Dutch literature, from 35% to 75% [4, 13]. Part of this variation can be explained by differences in measurement instruments and measurement procedures. For adequate benchmarking, uniformity in measurement procedures and instruments is needed. The aim of this study was to develop a minimum set of essential items to assess the quality of telephone triage. This core set should be incorporated into existing measurement instruments or can be used as a separate instrument.

## Methods

### Setting

The study was performed in a convenience sample of four out-of-hours GP cooperatives spread across the Netherlands.

### Instrument development

A national survey among representatives of all 49 umbrella organisations of GP cooperatives in the Netherlands was performed to determine the most important aspects of telephone triage and to collect existing measurement instruments, with a response rate of 71.4% (*N* = 35). In an online questionnaire, representatives of the GP cooperatives (mainly managers or quality functionaries) summed up the five aspects they thought were most important to be incorporated in an instrument for measuring the quality of triage. Twenty-two topics were mentioned by more than one person. We gathered 13 measurement instruments, of which a substantial part were adapted versions of the HAAK-scoring instrument. [13] For the topics mentioned by more than one respondent, corresponding measurement items were identified in the 13 existing measurement instruments. This resulted in a list of 210 candidate items for inclusion in the minimum set. An expert panel consisting of six GPs and six triage nurses judged these items on importance, completeness and wording in three online questionnaire rounds and one face-to-face meeting. They also determined whether the items concern medical or communicational aspects of quality.

The instrument used in the pilot study consisted of 24 items about the telephone conversation: 13 medical aspects, ten communicational aspects and one regarding both types. The items were arranged in three phases: 1) intake (eight items), 2) triage and follow-up (nine items) and 3) finishing-off (two items). In addition, there were five general items. The instrument can be extended by a module on the quality of the conversation of the triagist with the GP (two items)) and a module on the quality of the registration in the patient record (six items). These two modules are not described in this paper.

Items are scored on a five-point scale from 0 to 4:

- 0 = Absent: item is incorrectly absent (it was necessary for the triagist to perform this action)
- 1 = Insufficient: item is performed insufficiently
- 2 = Moderate: item is not performed insufficiently, but also not sufficiently
- 3 = Sufficient: item is performed sufficiently, but there is room for improvement
- 4 = Good: item is performed optimally; there is no room for improvement

For some items, there is the option ‘not applicable’. This option applies only if the item is justly absent. We chose not to give score 4 for justly absent items, because then the content or context of the call could influence the score (for example, calls for relatively severe health problems, resulting in a face-tot face contact with the GP (clinic consultation or home visit) would get score 4 on item 15 for the absence of self-care advice, while calls that are handled by the triagist (on telephone) have a lower chance to get the highest score). In addition, only for the validation of the instrument, overall grades for the impression of the quality of the medical content and communication of the triage were included in the instrument (two items). The measurement scale of the grades ranged from 1 (poor) to 10 (excellent).

The instrument was named KERNset, meaning the core (in Dutch: “kern”) set of items necessary to be included in an instrument for measuring the quality of telephone triage. An important document supplementary to the KERNset is the user manual, developed to aid the assessment and the interpretation of the items. In addition, there is a measurement procedure protocol including criteria for sampling and the background of the raters. All items of the instrument are shown in Table 1.

**Table 1:** Items in the questionnaire, distribution of scores, intra class correlation and percentages of agreement between external raters per item

	Score (%)						Inter-rater agreement (%)		ICC
	0	1	2	3	4	N/A	Complete	Reasonable	
	N = 342						*N* = 114	N = 114	N = 114
PHASE 1 INTAKE (item 1–8)							48.0	74.3	.16
Collect personal and residence information									
1. Gathers, at an appropriate time, the personal and residence information (c)	1	3	10	18	64	3	48.2	78.9	.20
ABCDE-check *(vital signs)*									
2. Asks to speak to the patient (c/m)	17	3	5	2	12	61	78.1	86.0	.70
3. Makes the right choice whether or not to perform the ABCDE-check (m)	13	12	13	14	49	–	36.8	65.8	-^a^
4. Checks the ABCDE-criteria adequately and draws the right conclusion (m)	11	14	7	11	9	49	40.4	57.9	.11
Open orientation									
5. Gives caller sufficient time to describe the situation (c)	0	2	12	23	63	–	45.6	84.2	.16
Medical problem and help request									
6. Asks for the medical problem and its development (m)	0	4	13	30	53	–	36.8	86.0	.24
7. Determines and explicitly states the help request (c)	26	8	10	16	40	–	47.4	71.9	.06
Complaint (in triage system)									
8. Selects *(in the triage system)* the appropriate complaint which yields the highest urgency (m)	5	8	9	11	67	–	50.9	64.0	.15
PHASE 2 TRIAGE AN FOLLOW-UP (item 9–17)							50.9	73.5	.27
Questioning according to triage methodology									
9. Asks at least the essential questions belonging to the specific complaint (m)	4	9	17	30	40	–	36.0	82.5	.27
10. Works according to the triage system of the GP cooperative (m)	1	4	3	12	21	61	61.4	62.3	.19
History and medication									
11. Asks the relevant questions with regard to history and medication (m)	19	6	5	12	53	5	52.6	81.6	.57
Urgency estimation									
12. Recognizes changes in the status of the patient and reacts adequately (m)	2	3	1	2	2	91	85.1	86.8	-^a^
13. Makes an appropriate urgency estimation (m)	3	13	13	16	56	–	37.7	65.8	.27
Follow-up action									
14. Chooses the right follow-up action (m)	1	9	10	14	66	–	48.2	73.7	.16
15. Gives the right (selfcare-) advice (m)	5	5	5	9	28	47	53.5	66.7	-^a^
16. Gives the right safety net advice about how to act in case of a change in the situation (m)	21	8	6	13	33	20	43.0	64.9	.10
17. Provides concise information which can be clearly understood by the caller (c)	0	3	11	23	64	–	40.4	77.2	.30
PHASE 3 FINISHING-OFF (item 18–19)							25.4	60.5	.15
Check follow-up action									
18. Asks if the follow-up action is understood and feasible (c)	13	8	17	26	35	–	27.2	60.5	.15
19. Checks whether the caller agrees with the follow-up action and shows an open attitude if the caller does not agree (c)	10	9	18	25	37		23.7	60.5	.14
GENERAL (item 20–24)							36.9	72.5	.19
Structure									
20. Structures the conversation (c)	1	5	23	33	38	–	32.5	78.9	.10
21. Makes use of open and closed questions adequately (c)	1	8	18	32	42	–	31.6	79.8	.19
Summary									
22. Gives a summary at an appropriate time, verifies and adjusts the summary if necessary (c)	31	12	11	20	26	–	33.3	71.1	.27
Sympathize									
23. Pays attention to the experience of the caller (c)	12	10	15	21	30	12	24.6	52.6	.20
Consultation general practitioner									
24. Consults the general practitioner only if necessary (m)	4	2	6	9	79	–	62.3	79.8	.05
MEDICAL ITEMS							51.6	73.1	.19
COMMUNICATION ITEMS							39.3	72.9	.19
TOTAL							44.9	72.5	.19

^a^ICC could not be estimated

m = medical item; c = communication item

N/A = not applicable

### Data collection

A power calculation showed a sample size of 120 triage conversations would be sufficient for the study, based on the needed sample size for the estimation of the intra class correlation coefficient [14]. The four participating GP cooperatives were asked to provide a sample of 30 anonymous triage conversations from ten different triage nurses (three calls per triagist) from the period July–December 2013. Per triagist, the criteria for sampling were:

- One highly urgent (U1-U2 from Dutch triage system), one moderately urgent (U3-U4) and one low urgent call (U5; telephone advice only)
- Each call had a different reason for encounter
- Exclusion of unintelligible calls, prematurely terminated calls, call back contacts, administrative contacts (e.g. for a prescription)

Both triagists and calls were randomly selected using the RAND function in Excel. For each call in the sample, the urgency (U1-U5), reason for encounter and patient age was recorded. Six calls eventually did not meet the sampling criteria, so 114 calls were included in the analyses. Patient and GP cooperative identifiers were deleted from the audio files. The calls were uploaded via a secured and encrypted internet connection and data were treated in confidence.

The triage conversations were rated by a pool of eight raters who were employed at one of the four GP cooperatives (two per GP cooperative). Six raters were certificated triagists, one was a GP and one was a location manager. The raters had on average 5.5 years of experience in rating calls with one of the existing triage observation instruments (range 3–10 years) and they rated between 25 and 250 triage conversations yearly.

Each triage conversation in the study sample was judged by three raters: one internal rater (of the own GP cooperative) and two external raters (of two other participating GP cooperatives). The raters received training in the use of the KERNset by a professional trainer, consisting of a four-hour face-to-face meeting and home training with the option to ask questions by e-mailing the trainer.

The raters used a digital rating form with a link to the audio file. They performed the rating at home or at work. The external raters were blinded for the GP cooperative the call was taken in, but the internal raters were not, because they knew the (voices) of the triagists. There were two rating periods with an intermediate period of four weeks. Each rater scored 44 or 45 calls during the first period and six or seven of the same calls during the second period.

### Statistical analyses

Three measurement characteristics were analysed: distribution of responses per item (criterion for skewedness: ≥80% of the responses within one category), inter-item correlations (criterion for too high correlation: Pearson correlation coefficient > 0.70) and internal consistency (Cronbach’s alpha of communicational and medical dimension). Scores of ‘not applicable’ and items with *N* < 50 were not included in the last two analyses.

The inter-rater reliability was determined by calculating the percentage of complete agreement (exactly the same score) and reasonable agreement (difference of maximally one point on the five-point scale) between the two external raters on each item. In addition, the agreement between the internal and external raters was calculated and their mean scores were compared to examine if one type of raters systematically scored higher than the other. To determine the intra-rater reliability, we compared the scores on 52 calls that were rated twice by the same rater with an interval of four weeks. The intra-rater agreement was calculated on the level of the dimensions and phases in the KERNset.

To determine construct validity, we calculated the correlation (Pearson correlation coefficient) between the mean score on the communicational/medical items of the KERNset and the overall impression grade on communicational/medical quality of the triage conversation. Also, the percentage of agreement between the rating with the KERNset and the overall impression grade was determined using dichotomised total KERNset scores (<2.5 inadequate; ≥2.5 adequate) and dichotomised overall impression grades (≤6 inadequate; >6 adequate).

Intraclass correlation coefficients (ICC) were calculated as a measure of the ability of the instrument to distinguish between triagists (criterion for sufficient ICC was .15 [15]). ICCs were calculated per item, per phase and for the medical and communicational dimensions. Skewed items were excluded (≥ 80% in one category). Scores of ‘not applicable’ were considered as missings in the analyses. The ICCs were calculated on the mean scores of the three raters per call. If two or all three raters scored ‘not applicable’, the call was excluded from the analysis (missing). Finally, we inspected the distribution of the scores 0–4 to see if the items had enough room for improvement.

To be able to perform the above analyses, the five-point rating scale was treated as a continuous variable. This increases the understandability of the results and is justified by the rather equal distance between each set of subsequent categories. The results should however be considered as approximations. The analyses were performed using the statistical software package IBM SPSS 20. Results were considered significant at *p* < 0.05.

## Results

### Description of calls and triagists

The mean call duration was six minutes and 40 s (range 2 min. 26 s. to 16 min. 47 s.). Of the patients, 59.6% were female with a mean age was 41.8 years (range 0 to 97). Due to the sampling method, the reasons for encounter showed a large variety, with shortness of breath, abdominal pain, vomiting, chest pain and extremity trauma as the most frequent reasons for encounter. The urgency varied from highly urgent (U1-U2: 32.5%), moderately urgent (U3-U4: 34.2%) to low urgent telephone advice (U5: 33.3%).

All 40 triagists were female with a mean age of 41.4 years (range 21 to 62). Their mean working experience was 5.7 years (range 0 to 13). Their professional background was practice assistant (65%), nurse (32.5%) or other (2.5%).

### Measurement characteristics

#### Distribution of responses

Table 1 shows the scores on all calls. The distribution of responses to item 12 was highly skewed ‘Recognizes changes in the status of the patient and reacts adequately’: 91% of the calls scored ‘not applicable’.

#### Inter-item correlations

Table 2 shows items with a Pearson correlation coefficient of .6 or more. The correlation was higher than .7 for three combinations of items: item 3 and 4 (*r* = .80), 9 and 10 (*r* = .77) and 18 and 19 (*r* = .79). These pairs of items basically measure the same concept.

**Table 2:** Pearson correlation coefficients between the items^a^

Item 1	Item 2	Pearson *r*
3. Makes the right choice whether or not to perform the ABCDE-check	4. Checks the ABCDE-criteria adequately and draws the right conclusion	.80 (*N* = 175)
18. Asks if the follow-up action is understood and feasible	19. Checks whether the caller agrees with the follow-up action and shows an open attitude if the caller does not agree	.79 (*N* = 342)
9. Asks at least the essential questions belonging to the specific complaint	10. Works according to the triage system of the GP cooperative	.77 (*N* = 135)
13. Makes an appropriate urgency estimation	14. Chooses the right follow-up action	.67 (*N* = 342)
8. Selects *(in the triage system)* the appropriate complaint which yields the highest urgency	9. Asks at least the essential questions belonging to the specific complaint	.66 (*N* = 342)
6. Asks for the medical problem and its development	21. Makes use of open and closed questions adequately (c)	.62 (*N* = 342)
10. Works according to the triage system of the GP cooperative	8. Selects *(in the triage system)* the appropriate complaint which yields the highest urgency	.62 (*N* = 135)
6. Asks for the medical problem and its development	9. Asks at least the essential questions belonging to the specific complaint	.61 (*N* = 342)
10. Works according to the triage system of the GP cooperative	17. Provides concise information which can be clearly understood by the caller	.61 (*N* = 135)
9. Asks at least the essential questions belonging to the specific complaint	21. Makes use of open and closed questions adequately (c)	.60 (*N* = 342)

^a^Correlations <.60 and with N < 50 are not reported

#### Internal consistency

Cronbach’s alpha was .94 for the 14 medical items and .75 for the 11 communication items. Deletion of the item that belonged to both dimensions (2: ‘Asks to speak to the patient‘) resulted in a slight increase in the Cronbach’s alpha scores, namely .95 for the medical items and .78 for the communication items. (Not in Table).

### Reliability

#### Inter-rater reliability

Table 1 shows the agreement between the two external raters. The total percentage of complete agreement (exactly the same score) was 44.9%. In 72.5% there was reasonable agreement (difference of maximally one point on the five-point scale). The percentage of complete agreement was higher for the medical items (51.6%) than for the communication items (39.3%). Allowing one-point differences (reasonable agreement), the agreement within the medical domain (73.1%) was comparable to the communicational domain (72.9%). The raters most often completely agreed on the items in phase 1 (48.0%) and phase 2 (50.9%) and less often on the items in phase 3 (25.4%) and the general items (36.8%) .

The highest complete agreement scores (>60%) were found on item 12 ‘Recognizes changes in the status of the patient and reacts adequately’, item 2 ‘Asks to speak to the patient’, item 24 ‘Consults the general practitioner only if necessary’, and item 10 ‘Works according to the triage system of the GP cooperative’. The high agreement scores for items 12, 2, and 10 are largely caused by the fact that these items were scored as ‘not applicable’ in many calls. Excluding the items with many ‘not applicable’ scores, the highest reasonable agreement (>80%) was found on item 5 ‘Gives caller sufficient time to describe the situation’, item 6 ‘Asks for the medical problem and its development’, item 9 ‘Asks at least the essential questions belonging to the specific complaint’, and item 11 ‘Asks the relevant questions with regard to history and medication’.

The lowest complete agreement scores (<30%) were found on item 18 ‘Asks if the follow-up action is understood and feasible’, item 19 ‘Checks whether the caller agrees with the follow-up action and shows an open attitude if the caller does not agree’, and item 23 ‘Pays attention to the experience of the caller’. The reasonable agreement scores were also low for these items.

#### Intra-rater reliability

Table 3 shows the intra-rater agreement after repeated assessment of the same call by the same rater. The total percentage of complete agreement was 55.1% and the reasonable agreement (difference of maximally one point) was 84.1%. Similar to the inter-rater agreement, there were differences between the phases, with the highest agreement scores in phase 1 and 2. The percentage of complete agreement was again higher for the medical items (64.6%) than for the communication items (59.1%). The reasonable agreement was 84.3% for the medical domain and 84.0% for the communicational domain.

**Table 3:** Percentage of agreement between repeated assessments per phase and dimension

	Agreement (%)	
	Complete	Reasonable
Phase 1: Intake (*N* = 416)	60.8	83.2
Phase 2: Triage and follow-up (*N* = 468)	63.9	86.5
Phase 3: Finishing-off (*N* = 104)	47.1	70.2
General (*N* = 260)	52.7	86.2
Medical items (*N* = 728)	64.6	84.3
Communication items (*N* = 572)	59.1	84.0
Total (*N* = 1248)	55.1	84.1

#### Internal versus external raters

The agreement between the internal and external raters was comparable to the agreement between the two external raters (Table 4).

**Table 4:** Percentage of agreement between internal and external rater per phase and dimension

	Complete agreement (%)		Reasonable agreement (%)	
	Internal vs external	*External* vs *external*	Internal vs external	*External* vs *external*
Phase 1: Intake (*N* = 416)	49.7	*48.0*	75.3	*74.3*
Phase 2: Triage and follow-up (*N* = 468)	49.2	*50.9*	69.5	*73.5*
Phase 3: Finishing-off (*N* = 104)	27.0	*25.4*	56.8	*60.5*
General (*N* = 260)	38.6	*36.8*	71.9	*72.5*
Medical items (*N* = 728)	51.3	*51.6*	72.3	*73.1*
Communication items (*N* = 572)	40.6	*39.3*	70.6	*72.9*
Total (*N* = 1248)	42.3	*44.9*	70.8	*72.5*

#### Differences between raters

Table 5 shows the mean scores on the KERNset per rater. The mean total scores varied from 2.42 (rater 4) to 3.30 (rater 6). The same pattern can be seen for the mean scores on medical and communicational items and this is not related to the quality of the rated calls, because the calls were randomly distributed to the raters. The raters of GP cooperative 3 and 4 generally gave higher scores (mean total score 3.14) than the raters of the other two GP cooperatives (mean total score 2.79 and 2.65).

**Table 5:** Mean score per location and rater (*N* = 342)

Location	Rater	Total	Medical items	Communication items
		Mean (SD)	Mean (SD)	Mean (SD)
1	1	2.95 (0.69)	2.93 (0.95)	2.81 (0.72)
	2	2.63 (0.59)	2.66 (0.75)	2.56 (0.70)
	*1 and 2*	*2.79 (0.66)*	*2.79 (0.86)*	*2.68 (0.72)*
2	3	2.88 (0.71)	2.87 (0.75)	2.90 (0.70)
	4	2.42 (0.76)	2.54 (0.95)	2.23 (0.74)
	*3 and 4*	*2.65 (0.77)*	*2.71 (0.86)*	*2.56 (0.79)*
3	5	2.98 (0.68)	3.23 (0.78)	2.67 (0.72)
	6	3.30 (0.56)	3.25 (0.66)	3.29 (0.62)
	*5 and 6*	*3.14 (0.64)*	*3.24 (0.72)*	*2.98 (0.74)*
4	7	3.14 (0.66)	2.96 (0.85)	3.23 (0.61)
	8	3.13 (0.40)	3.31 (0.44)	2.91 (0.50)
	*7 and 8*	*3.14 (0.55)*	*3.13 (0.70)*	*3.07 (0.58)*

### Validity

The Pearson correlation coefficient between the mean score on the *medical* items of the KERNset and the overall impression grade on *medical* quality of the triage conversation was .77. The correlation between the mean score on the *communicational* items and the overall impression grade on *communicational* quality was .73. Dichotomising the total KERNset scores and overall impression grades as adequate versus inadequate for each domain resulted in 83.9% agreement for the medical domain 80.7% for the communicational domain.

### Variation between triagists

The last column of Table 1 shows the median ICC per item, per phase and for the medical and communicational domains. In total, 19% of the variation on the items can be explained by differences between triagists. Phase 2 shows the highest median ICC (.27). Item 2 ‘Asks to speak to the patient’ and item 11 ‘Asks the relevant questions with regard to history and medication’ demonstrated the largest variation between triagists (ICC = .70 and ICC = .57 respectively) (Table 1).

Items with the largest room for improvement were item 2 ‘Asks to speak to the patient’ (excluding N/A, 65% of the scores ≤2), item 4 ‘Checks the ABCDE-criteria adequately and draws the right conclusion’ (61% of the scores ≤2) and item 22 ‘Gives a summary at an appropriate time, verifies and adjusts the summary if necessary’ (54% of the scores ≤2). Item 2 and 22 show relatively high ICCs in combination with relatively low scores on the KERNset.

## Discussion

### Main findings

Different instruments have been used for assessing the quality of telephone triage in GP cooperatives. These instruments focussed mainly on communicational aspects, and less on the medical quality of triage decisions. Moreover, because of the diversity in instruments, benchmarking between organisations was not possible. Our objective was not to create a new instrument, but we developed a minimum set of 24 items to be included in existing instruments to assess the quality of telephone triage.

The items appeared to be capable of measuring differences between calls; there was only one skewed item. Three pairs of items were strongly correlated. The internal consistency of the medical and communicational domain was high, indicating that the items within the domains all measure the same underlying construct.

The reliability of the KERNset in our pilot study was suboptimal. For the inter-rater reliability, the total percentage of complete agreement (exactly the same score) was 45%. Allowing one-point differences, the reasonable agreement was 73%. Intra-rater reliability was determined by repeated assessments. These showed a complete agreement of 55% and reasonable agreement of 84%. There were hardly any differences in agreement scores between the internal and external raters on the one hand and the external raters on the other hand. However, there were differences in strictness between the individual raters; mean scores varied from 2.4 to 3.3. The large differences in strictness between the raters partly explain the suboptimal inter-rater reliability. But the results also reflect the fact that the raters were used to different instruments and had to be retrained. The training they received was possibly insufficient to fully understand how to use the KERNset. A more intensive training and strict adherence to the user manual could improve the reliability.

The content validity of the KERNset is supported by its development, which relied on field opinions, items from existing instruments, and judgments of an expert panel. We found support for the construct validity by the high correlation between the general impression of the call and the items of the KERNset. In addition, the convergent validity has been verified in a parallel study in one of the participating GP cooperatives by comparing the scores on the KERNset with the scores of another telephone triage measurement instrument [16].

The KERNset appears to detect differences between triagists; on average 19% of the variation within items can be explained by differences between triagists. In addition, on most items there was a good variation in scores, indicating there is enough room for improvement of the quality of telephone triage.

### Strengths and limitations

We used an extensive procedure to develop the KERNset using existing instruments, field opinions, and expert opinions to determine which items define the quality of triage calls. We examined multiple aspects of the reliability and validity of the KERNset and tested its measurement characteristics.

We used raters who had experience with other instruments to assess the quality of telephone triage. They participated in a half-day training session supplemented by home training to learn the new instrument. The low inter-rater reliability indicates this training was insufficient.

To examine the variation between triagists we used three calls per triagist. More calls per triagist would have improved the reliability of the results, because the consequences of a potential selection of a call with a deviating content are larger in a small sample than in a large one.

Finally, we could not examine the ability of the KERNset to detect differences between GP cooperatives, because only four GP cooperatives were included in the pilot study.

### Implications for practice

The KERNset was slightly adjusted based on the results of our pilot study. Item 10 ‘Works according to the triage system of the GP cooperative’ was deleted because of high correlations with two other items. Also item 12 ‘Recognizes changes in the status of the patient and reacts adequately’ was deleted, because it was almost never applicable. Finally, item 2 ‘Asks to speak to the patient’ was included in the medical dimension, because it reduced the internal consistency of the communication al dimension and we wanted items to belong to one dimension only. The expert panel agreed on these adjustments.

Because of the suboptimal reliability of the KERNset, a group of experts has improved the descriptions in the user manual. The reliability should be further tested using this improved manual. Moreover, raters should receive more intensive training in the use of the KERNset.

Finally, the expert panel has recommended standards for sufficient quality for re-registration as a triagist: they have determined a minimum score on three essential items and minimum mean scores on the communicational and medical aspects.

## Conclusions

This study demonstrates that the KERNset can be used to assess the quality of telephone triage. The validity of the instrument is good and differences between calls and between triage nurses can be measured. A more intensive training for the raters and strict adherence to the user manual could improve the reliability. Future studies could examine the discriminative power between triagists and GP cooperatives.

## Abbreviations

GP: general practitioner
ICC: intraclass correlation coefficient
N/A: not applicable
SD: standard deviation
SE: standard error
SPSS: Statistical Package for the Social Sciences
